# Survey-based naming conventions for use in OBO Foundry ontology development

**DOI:** 10.1186/1471-2105-10-125

**Published:** 2009-04-27

**Authors:** Daniel Schober, Barry Smith, Suzanna E Lewis, Waclaw Kusnierczyk, Jane Lomax, Chris Mungall, Chris F Taylor, Philippe Rocca-Serra, Susanna-Assunta Sansone

**Affiliations:** 1 EMBL-EBI, Wellcome Trust Genome Campus, Hinxton, Cambridge, CB10 1SD, UK; 2 Institute of Medical Biometry and Medical Informatics (IMBI), University Medical Center, 79104 Freiburg, Germany; 3 Center of Excellence in Bioinformatics and Life Sciences, and Department of Philosophy, University at Buffalo, NY, USA; 4 Berkeley Bioinformatics and Ontologies Project, Lawrence Berkeley National Labs, Berkeley, CA 94720 USA; 5 Department of Information and Computer Science, Norwegian University of Science and Technology (NTNU), Trondheim, Norway; 6 NERC Environmental Bioinformatics Centre (NEBC), Mansfield Road, Oxford, OX1 3SR, UK

## Abstract

**Background:**

A wide variety of ontologies relevant to the biological and medical domains are available through the OBO Foundry portal, and their number is growing rapidly. Integration of these ontologies, while requiring considerable effort, is extremely desirable. However, heterogeneities in format and style pose serious obstacles to such integration. In particular, inconsistencies in naming conventions can impair the readability and navigability of ontology class hierarchies, and hinder their alignment and integration. While other sources of diversity are tremendously complex and challenging, agreeing a set of common naming conventions is an achievable goal, particularly if those conventions are based on lessons drawn from pooled practical experience and surveys of community opinion.

**Results:**

We summarize a review of existing naming conventions and highlight certain disadvantages with respect to general applicability in the biological domain. We also present the results of a survey carried out to establish which naming conventions are currently employed by OBO Foundry ontologies and to determine what their special requirements regarding the naming of entities might be. Lastly, we propose an initial set of typographic, syntactic and semantic conventions for labelling classes in OBO Foundry ontologies.

**Conclusion:**

Adherence to common naming conventions is more than just a matter of aesthetics. Such conventions provide guidance to ontology creators, help developers avoid flaws and inaccuracies when editing, and especially when interlinking, ontologies. Common naming conventions will also assist consumers of ontologies to more readily understand what meanings were intended by the authors of ontologies used in annotating bodies of data.

## Background

A wide variety of ontologies, controlled vocabularies, and other terminological artifacts relevant to the biological or medical domains are available through open access portals such as the Ontology Lookup Service (OLS) [1], and the number of such artifacts is growing rapidly. One of the goals of the Open Biomedical Ontologies (OBO) Foundry initiative [2] is to facilitate integration among these diverse ontologies. However, such integration demands considerable effort and differences in format and style can only add obstacles to the execution of this task [3]. The heterogeneity within the set of existing ontologies derives from the use of diverse ontology engineering methodologies and is manifest in the adoption by different communities of Description Logic, Common Logic, or other formalisms. The spectrum of syntaxes used to express these formalisms, such as the Web Ontology Language (OWL) or the OBO format, and the commitment of individual communities to conceptualist or realism-based philosophical approaches are also contributing factors.

Here we focus on issues of nomenclature [4], and specifically on the naming conventions used for labeling classes in ontologies, which are an additional contributing factor to the problem of heterogeneity. Even in this relatively straightforward area, no conventions have achieved broad acceptance (see survey section below).

The lack of naming conventions or their inconsistent usage can impair readability and navigation when viewing ontology class hierarchies. We believe that clear and explicit naming becomes of even greater importance when interlinking ontologies (for example via owl:import, obo dbxref and other referencing and mapping statements [5], or when ontology engineers need to collaborate with external groups to align their ontologies and to ensure effective maintenance of modularity).

While other sources of diversity are tremendously complex and challenging, it is our belief that establishing a set of naming conventions for the OBO Foundry is a tractable goal, particularly if those conventions are based on lessons drawn from pooled practical experience and targeted surveying.

There is of course no shortage of initiatives for the development of specifications and standards tackling naming [6-9]. However, where naming conventions have been developed, widespread application has been hampered by several factors, most notably domain specificity, document inaccessibility and format dependency. A comprehensive survey of existing naming convention documents can be found at the dedicated OBO Foundry naming conventions website [10].

### Domain specificity

One significant obstacle to common adoption is that many of the proposed conventions are domain-specific and not generally extendible to other fields; for example, the Human Genome Organization (HUGO) nomenclature [11] is restricted to gene names. Other conventions refer only to entities occurring within programming languages [12] or to the naming of natural language documents [13].

### Document inaccessibility

A second obstacle relates to poor documentation. A naming convention whose documentation is unclear, or is dispersed in multiple documents or document sections, artificially constrains its own chances of acceptance. This is the case with the BioPAX manual [14], which is in addition overly tool-centric in that it addresses only Protégé-OWL issues. Another deficiency is the commercial or semi-proprietary nature of conventions such as the International Organization for Standardization (ISO) standards [15]. Many of these proposed conventions also impair access through information overload, there being around forty ISO documents addressing naming issues alone. Other naming conventions are described only implicitly and via unintuitive search attributes, or are not available on-line, making access difficult.

### Format and implementation dependency

Sometimes only certain naming issues are tackled by a naming convention – usually those most germane to a particular format. The Gene Ontology (GO) Editorial Style Guide [16] for example, is of limited coverage and applicability, as it is embedded in an OBO-format specific document. The ANSI/ISO Z39.19-2005 Standard [8] is applicable only to terms organized in an *is-a *hierarchy without relations and therefore lacks proper conventions for representing ontological classes and properties in semantically complex ontologies.

In the case of the Ontology Engineering and Patterns Task Force of the Semantic Web Best Practices and Deployment working group [17], the guidelines are restricted to the OWL format and are dispersed throughout many documents and document sections.

To overcome this diversity and fragmentation members of the OBO Foundry and of the Metabolomics Standards Initiative (MSI) ontology working group [18] have set up an infrastructure group that is attempting to:

• collect, review and compare existing naming conventions

• distill universally valid conventions that can be implemented in both the OWL and OBO formats, and conceivably also in other formats

• engage in discussion with other groups concerned with nomenclature standardization in order to establish a forum for coordinated advance

• create a single common guideline document to serve as a common resource for the OBO Foundry and associated initiatives.

In this communication we present the preliminary results of a survey of the naming conventions applied by ontology groups listed under the OBO Foundry, together with an initial set of what we believe are robust conventions for formulation of terms in ontologies and a list of open issues that need to be resolved in the future.

## Results

### Survey

To determine the sources of heterogeneity in naming and to initiate a discussion among the ontology groups associated with the OBO Foundry, we carried out a survey. The goal was to allow us to:

• catalog the naming conventions that these groups currently apply

• learn about existing sets of documentation for the various naming conventions cataloged

• assess special requirements regarding the naming of entities in the context of various biological domains

• discover issues not yet addressed by our proposed conventions to determine future needs.

The survey was conducted by contacting the custodians of the 66 OBO ontologies (as of November 2007) either by email or telephone. Each respondent then received a questionnaire that was divided into four parts, covering:

1. Ontology engineering process and level of awareness of the OBO Foundry

2. Current practice in naming entities and documentation thereof

3. Implementation of different name categories

4. Questions on particular naming conventions

The full questionnaire, the complete set of answers and the consolidated results are available from the OBO Foundry wiki [10]. For more information on the survey results and list of participants see the Additional file 1: SurveyResults.zip.

### Naming Conventions

Our proposed set of naming conventions, founded on the survey results, is summarized in Table 1. In further discussions, we refer to the entities of which an ontology consists (in some circles these are called classes and relations) as its representational units [19]. A representational unit can be accompanied by one or more synonymous names of different categories. Any type of name that is chosen to be displayed in the hierarchy is called 'display name' (called 'browser key' in Protégé). Where the form of that name is controlled by a set of explicit rules we refer to it as a 'formal name'. To ensure that the conventions proposed here are expressed unambiguously we employ the following additional name categories, which we hope will also have general utility:

**Table 1:** The initial set of OBO Foundry naming conventions

***1. Be clear and unambiguous***			
**Naming Convention**	**Description**	**Example**	**Effect**

**1.1 Use explicit and concise names**	Keep names short and memorable, but precise enough to capture the intended meaning. Keep names linguistically correct and intuitively meaningful to human readers. Articles should be omitted.	'wall of esophagus', 'physical part' instead of 'the wall of the esophagus', 'distinct identifiable physical part'	Faster term recognition
**1.2 Use context independent names**	Apply names that are self-explanatory and understandable even when viewed outside of the immediate context of the ontology. Avoid truncated names and colloquialisms. In names, capture inherent and intrinsic characteristics rather than asserted and extrinsic characteristics. Avoid using names for non-role entities that refer to roles the entity referred to may potentially play in a particular context at a particular time. Capture product names as they are, but render them intelligible adding contextual information: [company name] + [product name] + [product type] (usually the superclass name). Additional information like the legal status of a company (e.g. Corp. or Inc.) should be omitted.	'NMR magnet' 'chemotherapy' and '1 ml pipette tip' instead of 'magnet', 'chemo' and 'blue pipette tip'. Use 'Bruker US 2 NMR magnet' instead of 'US 2'	Increases precision in the interpreted meaning. Helps string matching. Faster term recognition
**1.3 Avoid taboo words**	Affixes reflecting epistemological claims e.g., words that indicate types of representational units should be avoided in name.	'protocol' instead of 'protocol class'	Faster term recognition, redundancy reduction
**1.4 Avoid encoding administrative metadata in names**	Administrative metadata, e.g., a class' status and version should be factored out of the name and into suitable separate representational units	'protocol' instead of 'protocol (definition incomplete)'	Increases precision in the interpreted meaning

**2. *Be univocous***			

**Naming Convention**	**Description**	**Example**	**Effect**

**2.1 Use univocous names and avoid homonyms**	Names should have the same meaning on every occasion of use and refer to the same types of entities in reality. Homonyms, ambiguous terms that share the same spelling but have many different meanings, are to be avoided as part of editor-preferred names. Use terms with fewest possible amount of homonyms in building names	'protocol collection' instead of 'protocol set' for a plurality of protocols (store the latter as synonym), 'parameter adjustment' instead of 'parameter setting' for the act of setting parameters	Increases precision in the interpreted meaning. Faster term recognition
**2.2 Avoid conjunctions**	Words that are used to join other words, such as the logical connectives 'and' and 'or' should be avoided in names as they can introduce ambiguity and may hamper inference by causing excessive branching. The same applies to qualifiers such as 'in some cases'	In 'anatomic structure, system or substance' it is not clear whether the adjective "anatomic" is restricted to "structure" or extends also to "system" and "substance". In the first case the substances 'drug' and 'chemical' would be classified under this class, otherwise not.	Increases precision in the interpreted meaning
**2.3 Prefer singular nominal form**	Use singular names throughout. Where plurals need to be captured, e.g. when one instance of the plural class represents a plurality itself, consistently use explicit plural indicating postfixes as part of the class names, e.g. use 'aggregate', 'collective' or 'population' consistently, but only as applicable.	'pair of lungs', 'population' instead of 'lungs', 'people collection'	Increases precision in the interpreted meaning, helps string matching
**2.4 Use positive names**	Avoid use of negations in formulating names. Avoid complements and negative names like 'non-separation device' because logically this will include everything in the universe that is not a separation device. The absence of a characteristic is not a concise differentiating criterion. Do not represent the absence of a characteristic (e.g. wing) as the presence of the non-existence of a characteristic, e.g.: 'wing' has_status "absent".	Avoid 'non-linear model'	Increases precision in the interpreted meaning
**2.5 Avoid catch-all terms**	Avoid 'rag-bag' words that do not designate natural kinds. The existence of classes is not dependent on our biological knowledge	Avoid 'unlocalised', 'unknown', 'unclassified'	Increases precision in the interpreted meaning

**3. *Reduce string variance***			

**Naming Convention**	**Description**	**Example**	**Effect**

**3.1 Recycle strings**	Word compositions should be constructed in a consistent manner, rather than using para-synonymous strings interchangeably. When creating compound names re-use strings as they occur in names of entities already defined elsewhere in this or in other ontologies	'x part of process', 'y part of process' instead of 'x component of process', 'y portion of process'	Helps string matching Eases cross product generation
**3.2 Use genus-differentia style names**	Class names should reflect the differentia that distinguishes the class from its parent class (modifiers to the head word). These should be the same that are modelled explicitly, so that the name compounds can be mapped to representational units that are connected to that class.	'DNA microarray' is_a 'microarray' 'protein microarray' is_a 'microarray', where 'DNA' and 'protein' are the differentiae and are defined elsewhere	Eases cross product generation. Helps string matching
**3.3 Use space as word separators**	Use the bar space (' ') character as word separator, just as it would normally appear in the language of choice. Where use of the bar space is not allowed by the type of representational unit in use to store a name, the underscore ('_') should be used instead. Camel case should not be used as a means of word separation.	'DNA microarray',' 'pH value' instead of 'DNA_microarray', 'pHValue'	Faster term recognition Helps string matching
**3.4 Expand abbreviations and acronyms**	Spell out abbreviations and acronyms and capture truncated versions as synonyms. Acronyms that result in expressions that have other meanings should be avoided. Widely known acronyms (anacronyms) such as DNA and LASER can be used.	'high resolution probe' instead of 'HRP' or 'high res. probe.'	Faster term recognition, Increases precision in the interpreted meaning. Helps string matching
**3.5 Expand special symbols to words**	Special symbols and foreign language letter characters should be spelled out.	'degree Celsius', 'alpha helicase', 'carbon 14' instead of '°C', 'α helicase', 'C14'	Helps string matching

**4. *Typography***			

**Naming Convention**	**Description**	**Example**	**Effect**

**4.1 Prefer lower case beginnings**	Don’t enforce dogmatically, but use lower case beginnings for class and property names. Capture names just as they would appear in normal English written text, i.e. where acronyms and proper nouns cannot be avoided in names they should be capitalized.	Use ‘microarray’, ‘DNA microarray’, ‘pH value’, ‘Golgi apparatus’	Faster term recognition

**4.2 Avoid character formatting**	Use plain ASCII format to keep names as computationally pliant as possible. Subscripts, superscripts and accents should be avoided.	'Sigma-Aldrich' instead of 'Σ-ALDRICH™ "	Helps string matching

• **editor-preferred name**: A formal name used by the ontology's developers and adhering to their guidelines and naming conventions. Editor-preferred names are primarily constructed to aid those building and manipulating an ontology and should therefore be specified as the display name during ontology editing. The editor-preferred name for the Foundational Model of Anatomy (FMA) class FMA:3862 is 'Anterior interventricular branch of left coronary artery'.

• **user-preferred name**: An informal name chosen to meet the expectations of an end user community. Usually this would be the name most frequently found in the literature of the relevant domains, which can *inter alia *serve as an intuitive, queryable attribute for end users searching for data sets in a repository. The user-preferred names from FMA for FMA:3862 is 'Left anterior descending branch of left coronary artery'.

• **short name**: A very short name that is useful when displaying large, dense graphs (whose nodes are classes and whose edges are relations). A short name from FMA for FMA:3862 is the acronym 'LAD'.

Further types of names can be distinguished, such as 'lexical variant' (including abbreviations and acronyms), 'phonetic variant' and 'foreign language translation'. The one rule that governs all these name categories is that they all must be exact synonyms. Since Protégé and OBO Edit do not deal with external lexical formats in an integrated way, we recommend storing lexical variants in the ontology itself to make them immediately accessible e.g. when mapping ontologies and identifying homonyms.

The lack of defined name categories in the available representation languages has been recognized by the Ontology Task Force of the W3C Semantic Web Health Care and Life Sciences Interest Group [7] and the lack of clear guidance on which kind of name the representation language idioms rdfs:label (OWL) and term name (OBO) should contain, has contributed significantly to the current heterogeneity in naming between ontologies. Our minimum recommendation is to assign an editor-preferred name, to which all of the naming conventions described in Table 1 should be applied, and one or more user-preferred names, which are less controlled and chosen to match end user expectations and usage frequency. The utility of having separate editor- and user-preferred names is exemplified by the response to question 4.1.2 in our survey by the developers of the Drosophila development ontology where they describe the balance they attempt to strike between making names explicit, keeping them concise and avoiding straying too far from community usage.

## Discussion

Naming conventions for ontology engineering do not necessarily apply to other domains. For example, our recommendation "1.2 Use context independent names" (see Table 1) will not make sense in the domain of database schemata or object-oriented programming. Terms from ontologies can be used in annotations outside the ontological context, whereas a java class is always situated in a class library hierarchy and embedded in code, providing its full context and therefore its name does not need to be fully explicit. However, general naming conventions such as "1. Be clear and unambiguous" and "2. Be univocal" can be applied in database schema generation, class naming in object oriented programming, natural language generation, even Wikipedia article naming. Formulation of universally applicable naming conventions in the bio-ontology space is no easy task due to the multidimensional complexity of the area, deriving not least from its intrinsically interdisciplinary character. Therefore, although we have carried out a comprehensive survey of existing naming convention documents in different domains [10], we have deliberately confined ourselves here to considering the needs of the OBO Foundry community.

### Exceptions

When conventions have been established their application may be non-trivial, not least because of the exceptions which different groups will want to make to given rules. In cases where the conventions cannot be strictly applied, common sense should be used. Here we describe some situations of this sort highlighted by our survey.

#### Positive names (see 2.4 in Table 1)

The responses to question 4.8.1 showed that most groups already try to avoid negative names and names containing expressions such as 'without' or 'excluding'; yet nearly half of the survey respondents still found examples of negative names in their ontologies. It seems it can be difficult to decide when a term is negative; e.g., 'unhealthy', 'immaterial anatomical entity', 'nonlinear transformation', 'inorganic' and 'rotenone-insensitive'. The difficulty in defining the criteria for 'negative' indicates that the convention cannot be enforced strictly, but we hold that it is nonetheless a valuable guideline. Further, we recommend that explicit exclusions should not be made within names; e.g., as in 'hydrolase activity, acting on carbon-nitrogen (but not peptide) bonds, in cyclic amides' (GO:0016812).

#### Word separator (see 3.3 in Table 1)

We recommend the use of white space as separator in editor-preferred names. A consequence of the default behaviour of the Protégé 3.x Editor is that it encourages the use of the rdf:ID field to capture class names. Since this field can't contain spaces, developers using Protégé often use the underscore as a word separator. This can be cured by avoiding use of the rdf:ID field to record editor-preferred names and to use instead the rdfs:label field.

#### Expand Abbreviations (see 3.4 in Table 1)

When an abbreviation or acronym becomes more commonly used in everyday language than its full name, for example 'LASER', then it should be used as the name, with its expanded name captured as a synonym. In other words, usage frequency can take precedence over the rule of acronym avoidance.

#### Special character formatting and symbols (see 3.5 in Table 1)

The survey revealed that ontologies dealing with chemicals and using the IUPAC nomenclature need to apply character formatting to their names for purposes of semantic disambiguation. In ChEBI for example the full chemical name is represented with unrestricted character formatting, for example: CHEBI 30666: bis [tricarbonyl(η^5^-cyclopentadienyl)molybdenum](Mo-Mo). Since character formatting is not supported by most ontology editors and languages, the groups involved often develop specific tools to meet their requirements. For this reason ChEBI and the Systems Biology ontology have developed front ends built on top of relational databases to manage their ontologies. Defined character transformation rules can be used to encode special formatting for example as has been done by the Biological Imaging Methods Ontology, which uses [] for superscripts and [[]] for subscripts. In general these should be avoided.

### Benefits and applications

The application of common naming guidelines brings the following benefits:

• enhance communication between geographically dispersed developers

• simplify stand-alone ontology development and help in subsequent administration tasks

• simplify ontology networking; e.g., importing and using classes from external ontologies or imported ontology modules

• increase the accessibility and exportability of terms, facilitating re-use and reducing redundant development.

By increasing the robustness of ontology class names, a standard naming convention will:

• support the manual and automated integration (i.e., comparison, orthogonality-checking, alignment and mapping) of terminological artifacts

• facilitate access to ontologies through meta-tools such as the NCBO BioPortal by reducing the diversity with which these tools have to deal, thus reducing the burden on tool and ontology developers alike

• increase the robustness of context-based text mining for automatic term recognition and text annotation.

The proposed set of conventions is currently being applied by the Ontology for Biomedical Investigation (OBI) project [20] and by the Proteomics Standards Initiative (PSI) [21] and MSI ontology working groups. An example that illustrates how syntactic normalization enhances readability and navigability of the OBI ontology class hierarchy can be found on the OBO Foundry wiki [10].

The usefulness of design principles in general and naming conventions in particular increases considerably when they are supported by ontology editing tools [22]. In particular, tools should check for compliance to such conventions and provide the functionality not only to enforce, but also to exploit, convention-based naming patterns. We are pleased to observe that implementations of such functionality have already begun to appear. For example, in the OBO Edit 2 tool [23] redundant class names are indicated and users can also define their own verification checks by specifying filters and error messages that will be displayed for each name that matches (or fails to match) the conventions defined. This verification system can serve as a framework upon which to build robust checks for conformity to naming conventions, either as a built-in OBO Edit module or as externally provided plug-ins (John Day-Richter personal communication). Also tools such as OBOL that use the lexical information in class names are already being applied to find inconsistencies within and between labels, and to aid ontology integration and ontology engineering in general through the methodology of cross-products [24].

Some aspects of what we propose here mirror features of so-called Constrained Natural Languages, CNL [25]. In particular, defined restrictions on the use of grammar and terminology can be found in CNL, and exploiting developments in this field could prove fruitful. However we must be careful not to be seen to be trying to impose too great a burden on ontology editors by attempting to require them to learn another full representation language. It is important to stress that having conventions for default names (using the editor-preferred name as display name) does not place restrictions on the use of less formal or colloquial names, which can and should still be captured as synonyms.

### Impact on GO

As the longest established ontology in the OBO Foundry, GO has already invested effort in establishing its own naming conventions, having formerly suffered under many of the common pitfalls in naming described in this paper, for example, the use of catch-all terms such as "unlocalized" and "molecular function unknown" [26]. Some of the recommendations outlined here have been inherited from the GO community, which in turn will move to include this whole set of naming conventions into the GO style guide. The impact on GO will certainly be positive, especially where it is used in combination with other OBO Foundry ontologies. For example, GO is considering changing to the context-independent name "cell nucleus" (as already used in FMA), instead of "nucleus" to distinguish it from "atomic nuclei" in ChEBI. The avoidance of conjunctions in term names will decompose terms like "actin polymerization and/or depolymerization", and the restriction to positive names will prevent or lead to the refactoring of terms like 'non-eye photoreceptor cell development' in GO.

### Open Issues

The surveying process reported in this paper has been informative, and has provided evidence to support the various conventions presented herein. Furthermore, several responders explicitly stated that the questionnaire made them aware of issues which they had not thought of previously; and in some cases went on to indicate other areas where they considered that conventions would be helpful, such as:

• A reference terminology that names the various kinds of representational units (e.g., illustrating the differences between 'type', 'class', 'term', 'concept' and 'universal'), thereby supporting unambiguous discussion of particular representational units [19].

• Conventions for other representational units, such as the names of relations, instances and identifiers. For example, OBI uses the identifier convention [group prefix] + [underscore] + [unique number] (e.g., 'OBI_0000016'); whereas BFO simply uses a 'meaningful string' (e.g., 'IndependentContinuant'). In addition, relations do not have numeric identifiers, which should probably be changed as these representational units, like classes, undergo changes and updates.

• A formalism is needed for naming and marking administrative 'helper' classes and metadata bins within ontologies. Until recently, non-ontological classes in OBI, such as 'unclassified' (OBI_200067), 'to_be_fixed' (OBI_334), 'ChEBI_objects' (OBI_336), 'PATO_quality' (OBI_302), 'collected_relations' (OBI_400132) could be found side-by-side with domain-level classes. These are now marked as helper classes by adding an underscore as prefix.

• Branch, module, file and namespace naming conventions should be investigated. This is also indicated by the recurring discussions on ontology naming conflicts on the OBO discussion mailing list.

• It needs to be investigated in how far certain conventions are dependent on the degree of formality of the representational artefact at hand. Conventions regulating name compositions [24] may only be applicable to semantically granular ontologies using relations, but not to taxonomies.

• Besides our universal conventions, specialized ones for certain ontological classes of high interest, usage and abundance should be collected and evaluated. Such classes referring to 'processes', 'instruments' or 'organisations' are also called 'Named Entities' in the field of text mining.

Although work on some of the above issues has already started, these open issues are of importance and will be tackled in a next round of guideline development by the OBO Foundry coordinators, in collaboration with the OBO Foundry ontology developers.

## Conclusion

The effective and efficient description of scientific information is the ultimate goal of this work. Mature, consensus-based conventions to guide ontology development are a crucial requisite for the achievement of this goal. We have presented an initial set of naming conventions primarily (but certainly not exclusively) for use in OBO Foundry ontologies. The justifications for the conventions presented were founded on answers from ontology editor practitioners gathered by means of a survey carried out within the OBO Foundry community.

The resulting set of conventions should be viewed as a primer, to be expanded and refined on the basis of input from practitioners. These conventions were discussed and approved by representatives of the OBO Foundry ontologies at the first OBO Foundry Summit meeting in July 2008 at the European Bioinformatics Institute (EBI), Cambridge, UK, funded by the UK's Biotechnology and Biological Sciences Research Council (BB/E025080/1) and the Elixir project . Further feedback will allow us to continue refining and ultimately to finalize this proposal at the second OBO Foundry Summit meeting in June 2009 at the EBI. As part of this iterative development process we will continue to engage with other efforts, particular those outside the OBO Foundry community such as the W3C Semantic Web Health Care and Life Sciences Interest Group and the Ontology Engineering and Patterns Task Force of the W3C Semantic Web Best Practices and Deployment working group.

## Authors' contributions

This work was largely informed by the requirements of the annotation projects lead by SAS and PRS, who coordinated this work. DS was the knowledge engineer who reviewed the existing conventions and with SAS, PRS, BS, SL, CM and JL designed the survey. WK, BS and PRS worked with DS in defining the appropriate terminology for describing the naming conventions. Contributions and critical reviews by all the authors, in particular PRS, CT, SL, BS and SAS, delivered the final manuscript. Authors read and approved the final manuscript

## Supplementary Material

Additional file 1 **Surveying naming conventions within OBO Foundry ontologies**. This SurveyResults.zip is a webpage presenting the results of the naming conventions survey that was carried out within the OBO Foundry ontologies. It contains diagrams and tables illustrating the answers to the survey's questions, as well as the discussion of these results. Click here for file
