# Supplementary material for: Survey-based naming conventions for use in OBO Foundry ontology development
Source: BMC Bioinformatics. 2009 Apr 27;10:125. doi: 10.1186/1471-2105-10-125 (PMC2684543; doi:10.1186/1471-2105-10-125)
Supplement: Additional file 1 — Surveying naming conventions within OBO Foundry ontologies. This SurveyResults.zip is a webpage presenting the results of the naming conventions survey that was carried out within the OBO Foundry ontologies. It contains diagrams and tables illustrating the answers to the survey's questions, as well as the discussion of these results. [file 1471-2105-10-125-S1.zip › SurveyResults/index.htm]

Survey miniwebsite


|  |  |  |  |  |  |  |  |  |  |  |  |  |  |  |  |  |  |  |  |  |  |  |  |  |  |  |  |  |  |  |  |  |  |  |  |  |  |  |  |  |  |  |  |  |  |  |  |  |  |  |  |  |  |  |  |  |  |  |  |  |  |  |  |  |  |  |  |  |  |  |  |  |  |  |  |  |  |  |  |  |  |  |  |  |  |  |  |  |  |  |  |  |  |  |  |  |  |  |  |  |  |  |  |  |  |  |  |  |  |  |  |  |  |  |  |  |  |  |  |  |  |  |  |  |  |  |  |  |
| --- | --- | --- | --- | --- | --- | --- | --- | --- | --- | --- | --- | --- | --- | --- | --- | --- | --- | --- | --- | --- | --- | --- | --- | --- | --- | --- | --- | --- | --- | --- | --- | --- | --- | --- | --- | --- | --- | --- | --- | --- | --- | --- | --- | --- | --- | --- | --- | --- | --- | --- | --- | --- | --- | --- | --- | --- | --- | --- | --- | --- | --- | --- | --- | --- | --- | --- | --- | --- | --- | --- | --- | --- | --- | --- | --- | --- | --- | --- | --- | --- | --- | --- | --- | --- | --- | --- | --- | --- | --- | --- | --- | --- | --- | --- | --- | --- | --- | --- | --- | --- | --- | --- | --- | --- | --- | --- | --- | --- | --- | --- | --- | --- | --- | --- | --- | --- | --- | --- | --- | --- | --- | --- | --- | --- | --- | --- | --- | --- |
|

## Surveying naming conventions within OBO Foundry ontologies

We present the results of a survey carried out to establish which naming conventions are currently employed by  Open Biomedical Ontology (OBO) Foundry  ontologies and to determine what their special requirements regarding the naming of entities might be.

### Questionnaire

The survey was conducted by contacting the custodians of the 66 OBO ontologies (as of November 2007) either by email or telephone. Each respondent then received a questionnaire, that can be downloaded here:
Media:NC\_survey.doc.

### Participants

The overall response rate has been excellent and the Table
1 lists the ontologies that have participated in this survey. Of the 66 OBO ontologies contacted, 42 questionnaires were returned. The apparent failure of some groups to respond to the questionnaire is due to one of the following reasons, documented in the 'Comment' column; (i) two or more artifacts belong to the same ontology (i.e., GO exists as three individual artifacts, but only one questionnaire was returned); (ii) the same editorial group (or subsets of the same group) is behind two or more ontologies; (iii) the ontology in question is being re-engineered and merged with one or more others; (iv) the ontology is no longer maintained (fortunately this is only true in one case). For these reasons the surveying process has also been quite challenging *per se*, but this is a natural consequence of the dynamic nature of the ontology development process.

**Table 1.** OBO ontologies that have participated in the survey.

|  |  |  |
| --- | --- | --- |
| **OBO ontology name** | **Prefix** | **Comments** |
| Animal natural history and life history | ADW | ADW is outdated, questionnaire filled for 'ETHAN’, the new ontology |
| Biological imaging methods | Fbbi |  |
| **Biological process**, Cellular component, Molecular function | GO, GO, GO | Same (or subsets of the) editorial group: one questionnaire filled |
| BRENDA tissue / enzyme source | BTO |  |
| C. elegans development | WBIs |  |
| C. elegans gross anatomy | WBbt |  |
| C. elegans phenotype | WBPhenotype |  |
| Cell type | CL |  |
| Chemical entities of biological interest | CHEBI |  |
| Common Anatomy Reference Ontology | CARO |  |
| Dictyostelium discoideum anatomy | DDANAT |  |
| **Drosophila development**, Drosophila gross anatomy, Fly taxonomy, FlyBase Controlled Vocabulary | FBdv, FBbt, FBsp, FBcv | Same (or subsets of the) editorial group: one questionnaire filled |
| eVOC (Expressed Sequence Annotation for Humans) | EV |  |
| Foundational Model of Anatomy ("Lite" version) | FMA |  |
| **Habronattus courtship**, Loggerhead nesting |  | Same (or subsets of the) editorial group: one questionnaire filled |
| **Human developmental anatomy**, **timed version**, Human developmental anatomy, abstract version, Mouse gross anatomy and development | EHDA, EHDAA, EMAP | Same (or subsets of the) editorial group: one questionnaire filled |
| Human disease | DOID |  |
| Mammalian phenotype | MP |  |
| Medaka fish anatomy and development | MFO |  |
| Microarray experimental conditions | MO |  |
| **Molecule role (INOH Protein name/family name ontology)**, Event (INOH pathway ontology) | IMR, IEV | Same (or subsets of the) editorial group: one questionnaire filled |
| Mosquito gross anatomy | TGMA |  |
| Mouse adult gross anatomy | MA |  |
| Mouse pathology | MPATH |  |
| Multiple alignment | RO |  |
| NCI Thesaurus | NCIt |  |
| **Ontology for biomedical investigations**, NMR-instrument specific component of metabolomics investigations | OBI, NMR | Same (or subsets of the) editorial group: one questionnaire filled |
| Pathway ontology | PW |  |
| Phenotypic quality | PATO |  |
| **Physico-chemical methods and properties**, Physico-chemical process | FIX, REX | Same (or subsets of the) editorial group: one questionnaire filled |
| **Plant environmental conditions**, Plant growth and developmental stage, Plant structure, Cereal plant development, Cereal plant gross anatomy, Cereal plant trait, Maize gross anatomy | EO, PO, PO,GRO, GRO, TO, ZEA | Same (or subsets of the) editorial group: one questionnaire filled |
| Protein domain | IPR |  |
| **Protein modification**, Protein covalent bond | MOD | Merged with MOD |
| Protein-protein interaction | MI |  |
| Proteomics data and process provenance | ProPreO |  |
| Sample processing and separation techniques | SEP |  |
| Sequence types and features | SO |  |
| Systems biology | SBO |  |
| Tick gross anatomy | TADS |  |
| Unit | UO |  |
| Xenopus anatomy and development | XAO |  |
| Zebrafish anatomy and development | ZFA |  |

### Response documents and results

The questionnaire responses received and a summary table, with answers normalized for comparison and quantification, can be downloaded from the  OBO Foundry wiki.

Beside a textual evaluation we have visualized the main results in Table 2, showing responses to queries that were to be answered in a yes/no manner and Figure 1, illustrating qualitative survey responses to selected questions that were stated in an open manner.

Of the 42 survey respondents, 14 stated that they had developed their own naming conventions (question 2.1, Table 2). A closer look revealed that most of the documented conventions were both limited in coverage and embedded in papers or general style guides. 18 responders reported that in every case where naming conventions were documented, the naming of classes was tackled (question 2.2, Table 2). 10 responders stated they have conventions for naming relations, 9 had conventions on class IDs, 8 on namespaces, 7 on the name/version of the ontology itself and 8 responders had conventions for instance names. Of the respondents that did not document their naming conventions, 13 were using the ‘GO style guide’ for general guidance (see question 2.3, Figure 1). Both the Ontology for Biomedical Investigations (OBI) and Proteomics Standards Initiative (PSI) looked to the Metabolomics Standards Initiative (MSI) naming conventions document for guidance. Ontologies dealing with chemicals (e.g., ChEBI) usually draw on the International Union of Pure and Applied Chemistry (IUPAC) conventions for naming of small molecules. Of the species-specific ontologies, the Medaka Fish Ontology draws on the Zebrafish ontology; the mammalian phenotype group re-uses conventions of the Phenotype, Attribute and Trait Ontology (PATO). In the protein domain, InterPro and the Molecular Role Ontology (MRO) use UniProt names.

The survey further revealed that, many respondents captured more than one class name category, including synonymous alternative names, abbreviations and acronyms, brand names and plural forms (question 3.1, Figure 1). 31 respondents stated they capture a ‘user-preferred name’; 15 stated they capture a ‘formal name’ adhering to defined naming principles, 11 captured a short name to be displayed in large graphical representations; and 5 responders captured broader / narrower / related names. 5 respondents captured database cross-references (dbxref), and only 3 responders stated they capture foreign language translations in their artifacts.

The need of a number of ontology initiatives for higher-resolution representations of name categories, evidenced by the results described above is addressed explicitly in the survey (question 3.3, Table 2). It reveals that a third of respondents wished for a more detailed treatment of this issue, and for distinct representational units to be made available for the classification of these name categories in ontology languages themselves. Another third of respondents had no opinion on this issue, while the remainder - mostly OBO format users - felt that current naming practice is satisfactorily addressed in their current formalisms. It is notable that the problem of synonym types has been recognized by the Ontology Task Force of the W3C Semantic Web Health Care and Life Sciences Interest Group, which asserts that heterogeneous naming, and a lack of harmonization in the way names are represented in ontology languages, forces the construction of more complex queries by users and hinders the development of generic user interfaces.

The need for higher resolution in the representations of name categories is further illustrated by the development by many groups of their own metadata schemes to govern naming (e.g., OBI and MSI metadata annotation properties).

**Table 2.**  The results of the survey questions that were to be answered in a yes/no fashion.  The comment field contains additional and qualitative information that was provided by some responders.

|  |  |  |  |  |
| --- | --- | --- | --- | --- |
| **Question** | **Yes** | **No** | **Nil** | **Comment** |
| 1.2. Have you started implementing the OBO Foundry principles and aligned your development process accordingly? | 24 | 9 | 9 | Of the Obo Foundry ontologies two stated they are not implementing all Foundry principles: Cell type ontology due to resource constraints and Chebi due to dependencies to external terminologies, e.g. IUPAC. Principle 6 (all terms have definitions) can not be fulfilled for all chemical entities due to the workload this would cause. Since formal IUPAC names represent a (semi-formal) definition, natural text based definitions seem not to be needed. |
| 2.1 Have you developed naming conventions within your ontology community? | 14 | 27 | 1 | Most of the documented conventions were both limited in coverage and of limited visibility (embedded in papers or general style guides). Find more details on the OBO Foundry naming wiki. |
| 2.3 (If your answer to 2.1 is negative) Have you re-used existing naming conventions from other ontology groups? | 23 | 9 | 10 | Most looked at the GO style guide for guidance. OBI and PSI looked to the MSI naming conventions document. Ontologies dealing with chemicals (e.g. ChEBI) usually draw on IUPAC conventions. Species-specific ontologies often looked at conventions already present in related species ontologies. Sometimes usable conventions were applied coming from multiple dispersed documents. |
| 3.3 Do you think there is a need to expand the expressivity of the ontology representation languages in order to provide more naming flexibility? | 14 | 14 | 14 | For the actual requirements regarding new name categories in an expanded representation, see Diagram box and the OBO Foundry naming wiki. |
| 4.2.2 Do your names contain defined strings that have a special defined meaning in each occurrence? | 13 | 26 | 3 | Re-occuring fixed strings mentioned were ‘sensu’ (3 times) and  ‘method’, ‘Methyl- xy’ and ‘xy- group’, ‘but not’ and cardinal\_part\_of' (each one time). Find more details on the OBO Foundry naming wiki. |
| 4.2.3 Have you developed any guidelines to create compound names? | 9 | 26 | 7 | Nearly a quarter of the responders have developed syntactic conventions on compound names, e.g. regulating word order or grammar. INOH-EVO applies a morphological convention that renders verbs into their nominal forms, e.g. ‘TAK1 phosphorylates NLK1’ becomes ‘Phosphorylation of NLK1 by TAK1’. |
| 4.5.1 Do you encode word forms consciously and in a consistent way within your names? | 23 | 6 | 13 | Most groups used the time neutral singular noun form for class names. Properties are mostly formulated in present tense form. |
| 4.8.1 Do you apply negative names such as ‘non-separation device’? | 10 | 28 | 4 | Most of the responders avoid ‘excluding’ names such as ‘non-separation device’, but a quarter of the responders found examples of negative names in their ontologies. |

**Figure 1.**  It
illustrates the survey responses to questions that
were stated in an open manner. Each diagram
lists the absolute numbers of OBO Foundry ontology curators that
mentioned a specific idiom in the query answer.

|


### Contribute

If you wish to comment the presented conventions or propose additional ones, please fill in the questionnaire Media:NC\_survey.doc or contribute directly to the Naming Conventions discussion wiki.


|  |
