# Supplementary figures and images for: Survey-based naming conventions for use in OBO Foundry ontology development
Source: BMC Bioinformatics. 2009 Apr 27;10:125. doi: 10.1186/1471-2105-10-125 (PMC2684543; doi:10.1186/1471-2105-10-125)

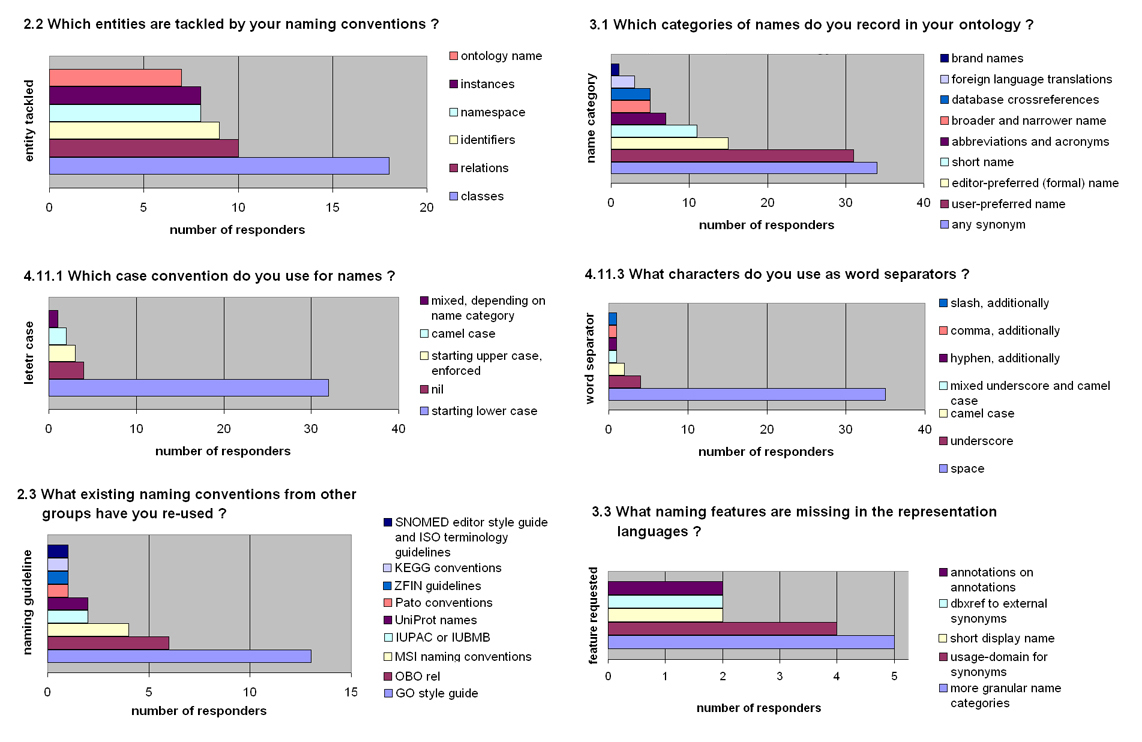

Supplement: Additional file 1 — Surveying naming conventions within OBO Foundry ontologies. This SurveyResults.zip is a webpage presenting the results of the naming conventions survey that was carried out within the OBO Foundry ontologies. It contains diagrams and tables illustrating the answers to the survey's questions, as well as the discussion of these results. [file 1471-2105-10-125-S1.zip › SurveyResults/SurveyResDiagramsSmall.jpg]
